# T-cell derived acetylcholine aids host defenses during enteric bacterial infection with *Citrobacter rodentium*

**DOI:** 10.1371/journal.ppat.1007719

**Published:** 2019-04-11

**Authors:** Valerie T. Ramirez, Dayn R. Godinez, Ingrid Brust-Mascher, Eric B. Nonnecke, Patricia A. Castillo, Mariana Barboza Gardner, Diane Tu, Jessica A. Sladek, Elaine N. Miller, Carlito B. Lebrilla, Charles L. Bevins, Melanie G. Gareau, Colin Reardon

**Affiliations:** 1 Department, of Anatomy, Physiology, and Cell Biology, UC Davis School of Veterinary Medicine, UC Davis, Davis, California, United States of America; 2 Department of Microbiology & Immunology, UC Davis School of Medicine, UC Davis, Davis, California, United States of America; 3 Department of Chemistry, UC Davis, Davis, California, United States of America; University of Toronto, CANADA

## Abstract

The regulation of mucosal immune function is critical to host protection from enteric pathogens but is incompletely understood. The nervous system and the neurotransmitter acetylcholine play an integral part in host defense against enteric bacterial pathogens. Here we report that acetylcholine producing-T-cells, as a non-neuronal source of ACh, were recruited to the colon during infection with the mouse pathogen *Citrobacter rodentium*. These ChAT^+^ T-cells did not exclusively belong to one Th subset and were able to produce IFNγ, IL-17A and IL-22. To interrogate the possible protective effect of acetylcholine released from these cells during enteric infection, T-cells were rendered deficient in their ability to produce acetylcholine through a conditional gene knockout approach. Significantly increased *C*. *rodentium* burden was observed in the colon from conditional KO (cKO) compared to WT mice at 10 days post-infection. This increased bacterial burden in cKO mice was associated with increased expression of the cytokines IL-1β, IL-6, and TNFα, but without significant changes in T-cell and ILC associated IL-17A, IL-22, and IFNγ, or epithelial expression of antimicrobial peptides, compared to WT mice. Despite the increased expression of pro-inflammatory cytokines during *C*. *rodentium* infection, inducible nitric oxide synthase (*Nos2*) expression was significantly reduced in intestinal epithelial cells of ChAT T-cell cKO mice 10 days post-infection. Additionally, a cholinergic agonist enhanced IFNγ-induced *Nos2* expression in intestinal epithelial cell *in vitro*. These findings demonstrated that acetylcholine, produced by specialized T-cells that are recruited during *C*. *rodentium* infection, are a key mediator in host-microbe interactions and mucosal defenses.

## Author summary

The nervous system is an active contributor to the regulation of immune responses. Prior studies have identified a unique CD4^+^ T-cell population that can relay signals from the sympathetic nervous system. These specialized T-cells express the enzyme choline acetyltransferase (ChAT) and produce acetylcholine (ACh). Release of ACh in response to neurotransmitters from the sympathetic innervation was previously shown to aberrant immune cell activation, reducing mortality during septic shock. Also, these CD4^+^ ChAT^+^ T-cells were previously found to control host-commensal interactions in naïve mice, but their role during enteric bacterial infection was unknown. Here we demonstrate that infection with *C*. *rodentium* induces ChAT^+^ T-cell recruitment and that expression of ChAT by this T-cell population significantly augments host defenses. These data support a diverse and expanding role of ACh in host immune responses.

## Introduction

The recently revealed degree of integration between the nervous and immune systems are remarkable [1]. While it is well accepted that neurotransmitters can act on immune cells to alter cell activation and consequently host immune response, recent evidence demonstrates that select immune cell populations not only respond but can also produce neurotransmitters. Among these immune cells are the CD4^+^ T-cells that express choline acetyltransferase (ChAT), the enzyme required for acetylcholine (ACh) biosynthesis [2–4]. These T-cells are crucial intermediaries between the nervous and immune system, functioning to relay neuronal signals and prevent aberrant immune cell activation. Neural inhibition of inflammation can inhibit innate immune cell function in preclinical models of inflammatory bowel disease [5], rheumatoid arthritis [6], ischemia reperfusion injury [7, 8], and post-operative ileitis [9]. Immune regulation in this pathway requires norepinephrine (NE) released from neurons to activate β2 adrenergic receptors (β2AR) on ChAT^+^ T-cells causing the release of ACh [2].

Mucosal immunity is crucial to restricting access of commensal and pathogenic bacteria to the host. Host defenses are comprised of overlapping mechanisms that bind, flush away, exclude, or kill pathogenic enteric bacteria [10]. These roles are in part fulfilled by differentiated intestinal epithelial cells (IECs) that not only act as a physical barrier, but also produce and release mucus [11], bactericidal antimicrobial peptides [12, 13], and free radicals such as nitric oxide (NO) that are bactericidal or bacteriostatic [14, 15]. Loss of these protective mechanisms can result in aberrant immune responses to otherwise innocuous commensal bacteria, increased mucosal inflammation, or susceptibility to infection. In addition, mucosal homeostasis and host-resistance to pathogens is dependent on composition of the intestinal microbiota, with bacterial species that can reduce, or enhance susceptibility to pathogens including *Citrobacter rodentium* [16–18]. Physiological processes that govern these mechanisms of host defense and host-bacterial interactions are therefore paramount to the health of the host.

In the gastrointestinal tract, ACh enhances mucosal protection by controlling IEC functions ranging from release of mucus and antimicrobial peptides to increasing ion and fluid secretion [12, 19, 20]. Together, these mechanisms of mucosal defense maintain homeostatic interactions between the host and commensal microbiota, while limiting access of pathogens such as *C*. *rodentium*. Although the source of ACh regulating these functions of IEC has long been attributed to ChAT^+^ secretomotor neurons within the gastrointestinal tract, we and others have previously described ChAT^+^ T-cells that serve as essential non-neuronal sources of ACh [2–4]. This unique source of ACh appears to participate in mucosal immunity and host commensal interactions. As evidence of this, conditional ablation of ChAT in T-cells was found to reduce production of antimicrobial peptides in the small intestine of naïve mice, and induce changes in the jejunal but not colonic microbiota composition [13]. Despite these key observations, the role of ACh released from specialized T-cells during enteric infection is unknown. With these issues in mind, we have used ChAT-GFP reporter mice, and conditional ablation of ChAT in T-cells to assess the role of T-cell derived ACh in host mucosal immune function during *C*. *rodentium* infection.

Using this approach, we have identified that ChAT^+^ T-cells are recruited to the colon during *C*. *rodentium* infection, and that conditional ablation of ChAT in T-cells significantly increases *C*. *rodentium* burden in the colon. This increased susceptibility to infection is due to decreased expression nitric oxide synthase isoform 2 in IEC, with ACh acting to enhance IFNγ-induced gene transcription.

## Methods

### Mice

Mice used in this study are on a C57BL/6 background and were originally purchased from Jackson laboratories (Bar Harbor, ME), including CXCR5-/-, ChAT-GFP (B6.Cg-Tg(RP23-268L19-EGFP)2Mik/J)), ChAT^f/f^ and LCK.Cre to establish a breeding colony. ChAT T-cell conditional knockout (cKO) mice were produced by breeding ChAT^f/f^ and LCK.Cre mice to generate LCK.Cre^-^ ChAT^f/f^ (WT) and LCK.Cre^+^ ChAT^f/f^ (cKO) mice. This breeding scheme permitted use of littermate cKO and WT controls. At 6–8 weeks of age, mice were gavaged with either LB, or *Citrobacter rodentium* (10^8^ CFU (colony-forming unit), strain DBS100, generously provided by Dr. Andreas Baumler). In a subset of experiments, colitis was induced by administration of dextran sodium sulfate (DSS, 3% v/v) in the drinking water for 5 days followed by normal water for 3 days as previously published [21].

### Ethics statement

All procedures were approved by the Institutional Animal Care and Use Committee at UC Davis under protocol number 20170 in accordance with the recommendations in the Guide for the Care and Use of Laboratory Animals. Mice were euthanized by CO_2_ asphyxiation followed by cervical dislocation according to American Veterinary Medical Association guidelines for collection of tissues.

### Flow cytometry

IEC and lymphocytes were isolated from the colonic lamina propria according to a standard protocol [22, 23]. In brief the colons from mice were removed, cut open along the mesenteric boarder, washed in PBS, before being place in Hanks buffered salt solution (HBSS) with EDTA (5 mM) to remove IEC. For experiments to ascertain the amount of NOS2 expressed by IEC, dissociated cells suspensions were incubated for 20 min with Fc blocking (CD16/32) antibody (TONBO Biosciences, San Diego, CA) and subjected to staining using Live/Dead aqua viability stain (ThermoFisher Scientific, Waltham, MA), anti- CD45-FITC (ThermoFisher Scientific) and -EpCAM-BV421 (BD Biosciences, San Jose, CA). IEC were then subjected to fixation and permeabilization using Fix/Perm buffer, and washed with permeabilization according to manufacturer’s instructions (BD Biosciences). Intracellular staining was then conducted using anti-NOS2-APC (ThermoFisher Scientific). Lymphocytes were isolated from colonic tissues subjected to removal of IEC as above, followed by digestion of 1 cm cut fragments in Collagenase IV (40 mg/ml, Sigma Aldrich). Released lymphocytes were incubated in RPMI media (10% FBS Pen/strep L-glutamine (ThermoFisher Scientific)) supplemented with PMA/Ionomycin (Cell Stimulation Cocktail 1X, eBioscience), with Golgi-Stop (BD Biosciences) for 4 hours. Samples were subjected to staining with Live/Dead Aqua (ThermoFisher Scientific), anti -CD3-BV605 (BioLegend, San Diego, CA), anti-CD4-APC (TONBO Biosciences), followed by fixation and intracellular staining for with anti -IL-22-PerCPeFluo710 (ThermoFisher Scientific), -IL-17A-PE (BD Biosciences), -IFNγ-PECy7 (ThermoFisher Scientific) and -GFP-AF488 (BioLegend). All samples were then acquired on a BD LSRII, and assessed using FlowJo (Treestrar, Ashland OR).

### Quantification of colonic *C*. *rodentium*

A 1 cm segment of colon was cut and placed into a pre-weighed microcentrifuge tube to determine tissue weight. Samples were homogenized in sterile PBS using a 5 mm sterile stainless-steel bead (Qiagen, Germantown MD) in a bead beater (Qiagen). Sample homogenates were then diluted in sterile PBS, and (100 μL) plated onto MacConkey agar plates, and colonies counted after 16 h of incubation at 37°C.

### Quantitative PCR

Analysis of gene expression was performed by quantitative real-time PCR (qRT-PCR) as previously described [3]. Briefly, a 1 cm long segment of colon was homogenized in Trizol (Invitrogen, Carlsbad, CA) using a 5 mm stainless steel bead in a bead beater (Qiagen). RNA was extracted as directed by manufacturer’s instructions, with isolated RNA dissolved in ultrapure H_2_O (Invitrogen). Synthesis of cDNA was performed using an iSCRIPT reverse transcriptase kit (Bio-Rad, Hercules, CA), and Real time qPCR was performed for the following targets using the indicated primer pairs from Primerbank [24]: *IL-1β* forward 5’-CTGTGACTCATGGGATGATGATG-3’, reverse 5’-CGGAGCCTGTAGTGCAGTTG-3’, *IL-6* forward 5’- TAGTCCTTCCTACCCCAATTTCC-3’, reverse 5’- TTGGTCCTTAGCCACTCCTTC-3’, *IL-17A* forward 5’- TTTAACTCCCTTGGCGCAAAA-3’, reverse 5’-CTTTCCCTCCGCATTGACAC-3’, *IL-22* forward 3’-ATGAGTTTTTCCCTTATGGGGAC-5’, reverse 3’-CTGGAAGTTGGACACCTCAA-5’, IFNγ forward 5’- GCCACGGCACAGTCATTGA-3’, reverse 5’- TGCTGATGGCCTGATTGTCTT-3’, *Tnfα* forward 5’-CCCTCACACTCAGATCATCTTCT-3’, reverse 5’-GCTACGACGTGGGCTACAG-3’, *NOS2* forward 5’-GTTCTCAGCCCAACAATACAAGA-3’, reverse 5’- GTGGACGGGTCGATGTCAC-3’, *Chi3L3* forward, CTCTGTTCAGCTATTGGACGC, reverse 5’- CGGAATTTCTGGGATTCAGCTTC-3’, *MRC1* forward 5’- CTCTGTTCAGCTATTGGACGC-3’, reverse 5’- CGGAATTTCTGGGATTCAGCTTC, *Retnla* forward 5’- CCAATCCAGCTAACTATCCCTCC- 3’, reverse 5’- ACCCAGTAGCAGTCATCCCA -3’, *Arg1* forward 5’-CTCCAAGCCAAAGTCCTTAGAG-3’, reverse 5’-AGGAGCTGTCATTAGGGACATC-3’, *Cxcl13* forward 5’- GGCCACGGTATTCTGGAAGC-3’, reverse 5’- GGGCGTAACTTGAATCCGATCTA-3’, *Ccl1* forward 5’- GGCTGCCGTGTGGATACAG-3’, reverse 5’- AGGTGATTTTGAACCCACGTTT-3’, *Ccl8* 5’- TCTACGCAGTGCTTCTTTGCC-3’, reverse 5’- AAGGGGGATCTTCAGCTTTAGTA -3’, *Ccl19* forward 5’- GGGGTGCTAATGATGCGGAA-3’, reverse 5’- CCTTAGTGTGGTGAACACAACA-3’, *Ccl21* forward 5’- GTGATGGAGGGGGTCAGGA-3’, reverse 5’- GGGATGGGACAGCCTAAACT-3’, *Defa3* forward 5'- GAGAGATCTGGTATGCTATTG-3', reverse 5'- AGCAGAGTGTGTACATTAAATG-3', *Defa5* forward 5'- TCAAAAAAGCTGATATGCTATTG-3', reverse 5'- AGCTGCAGCAGAATACGAAAG-3', *Defa20* forward 5'- GAGAGATCTGATATGCTATTG-3', reverse 5'- AGAACAAAAGTCGTCCTGAG-3', *Defa21* forward 5'- GAGAGATCTGATCTGCCTTTG-3', reverse 5'- CAGCGCAAAAAAGGTCCTGC-3', *Defa22* forward 5'- GAGAGATCTGATCTGCCTTTG-3', reverse 5'- CAGCGCAAAAAAGGTCCTGC-3', *Defa23* forward 5'- GAGAGATCTGGTATGCTATTG-3', reverse 5'- AGCAGAGCGTGTATATTAAATG-3', *Defa24* forward 5'- GAGAGATCTGGTATGCTATTG-3', reverse 5'- AGCAGAGCATGTACAATAAATG-3', *Defa26* forward 5'- ATTGTAGAAAAAGAGGCTGTAC-3', reverse 5'- AGCAGAGTGTGTACATTAAATG-3', *Itln1* forward 5'- ACCGCACCTTCACTGGCTTC-3', reverse 5'- CCAACACTTTCCTTCTCCGTATTTC-3', *Reg3g* forward 5'- CCTCAGGACATCTTGTGTC-3', reverse 5'- TCCACCTCTGTTGGGTTCA-3', *Lyz1* forward 5'- GCCAAGGTCTACAATCGTTGTGAGTTG-3', reverse 5'- CAGTCAGCCAGCTTGACACCACG-3', *Actb* forward 5’-GGCTGTATTCCCCTCCATCG-3’, reverse 5’- CCAGTTGGTAACAATGCCATGT-3’. *CIIta* forward 5’-TGCGTGTGATGGATGTCCAG-3’, reverse 5’-CCAAAGGGGATAGTGGGTGTC-3’, *Irf1* 5’-ATGCCAATCACTCGAATGCG-3’, reverse 5’-TTGTATCGGCCTGTGTGAATG-3’. Amplification and data acquisition were conducted using a QuantStudio6 (ThermoFisher Scientific). Data were analyzed using the delta delta CT method normalizing gene expression to *Actb* in each sample followed by normalization to experimental control sample.

### Analysis of short-chain fatty acids (SCFA) in fecal pellets by LC-MS

Fresh fecal samples from mice were collected and stored at -80°C until analysis. Pellets were extracted with nano-pure water (10 mg/mL) and gently agitated overnight at 4°C. The homogenized samples were centrifuged at 21,000 g for 5 min. Supernatants (100 μl) were transferred and centrifuged at 21,000 g again for 20 min. For each sample, 20 μl of the supernatant was mixed with 20 μl of 100 mM N-(3-Dimethylaminopropyl)-N0-ethylcarbodiimide hydrochloride (1-EDC HCl) (Sigma-Aldrich, cat. # E7750) in 5% pyridine (Sigma-Aldrich cat. # 270407) and 40 μL of 200 mM 2-Nitrophenylhydrazine (2-NPH) (Sigma-Aldrich, cat. # N21588) in 80% acetonitrile (ACN) (Sigma-Aldrich) with 50 mM HCl. The mixture was incubated at 40°C for 30 min. After reacting, 400 ml of 10% ACN was added to the solution. Then 1 μl of the solution was injected into an Agilent 6490 triple quadruple mass spectrometer for analysis. Chromatographic separations were carried out on an Agilent C18 stationary phase (2.1 x 50 mm, 1.8 um) column. Mobile phases were 100% ACN (B) and water with 10% ACN (A). The analytical gradient was as follows: time = 0 min, 10% B; time = 4.5 min, 10% B; time = 10 min, 35% B; time = 10.1 min, 85% B; time 11.6 min, 90% B; time 12 min, 90% B. Flow rate was 0.3 ml/min and injection volume was 1 μL. Samples were held at 4°C in the autosampler, and the column was operated at 40°C. The MS was operated in selected reaction monitoring (SRM) mode, where a parent ion is selected by the first quadrupole, fragmented in the collision cell, then a fragment ion selected for by the third quadrupole. Product ions, collision energies, and cone voltages were optimized for each analyte by direct injection of individual synthetic standards. The MS was operated in positive ionization modes with the capillary voltage set to 1.8 kV. Source temperature was 200°C and sheath gas temperature 200°C. Gas flow was 11 L/min, sheath gas flow was 7 L/min, and collision gas flow was 0.2 mL/min. Nebulizer pressure (nitrogen) was set to 25 psi. Argon was used as the collision gas. A calibration curve was generated using authentic standards for each compound.

### Histology

Colonic tissue specimens were fixed in 10% normal buffered formalin for 24 h prior to gradual dehydration in ethanol, embedded in paraffin and 6 μm thick cross sections were cut onto glass slides. Slides with tissue sections were de-paraffinized and rehydrated according to standard protocols, stained with hematoxylin and eosin to allow for evaluation of histopathology. Crypt lengths were measured using bright field microscopy on these sections with FIJI (*Fiji* Is Just ImageJ) [25], measuring at least 20 crypts per animal.

### Confocal microscopy

Slides with colonic tissue section were also used for confocal analysis with antibodies raised against specific proteins of interest according to standard protocols. In brief, after slides were de-paraffinized and rehydrated, antigen retrieval was performed in citrate buffer (10 mM, pH 6.0, 30 min., 95°C). After blocking in 5% BSA and normal donkey serum, samples were incubated in primary antibody overnight (16 h 4°C). Primary and secondary antibodies used are detailed in **Table 1.** Slides were washed extensively (3 x 5 mins) in TBS-tween20 and incubated in appropriately labeled secondary antibodies (Invitrogen) for 1 h at room temperature, washed, counterstained with DAPI in TBS-tritonX100 0.1% v/v, washed and mounted in Prolong gold (Invitrogen). Staining using anti-mouse CDH1 (E-cadherin) was revealed using a mouse on mouse kit according to manufacturer’s instructions (Vector laboratories, Burlingame, CA). Slides were imaged on a Leica SP8 STED 3X confocal microscope with a 63X 1.4 NA objective. Areas larger than the field of view of the objective were acquired using a tiling approach, whereby adjacent images were acquired with a 10% overlap.

**Table 1:** Antibodies used for confocal microscopy.

**Target**	**Host**	**Source**	**Catalog No./clone**	**Dilution**	
CDH1	Rbt	ECM Biosciences	CP1921	1:400	
CDH1	Ms	ECM Biosciences	CM1681	1:400	
GFP	Goat	Rockland Immunochemicals	600-101-215	1:300	
CD3	Rat	Bio-Rad	CD3-12	1:100	
*Citrobacter koseri* (cross-reactive to *C*. *rodentium*)	Rabbit	Abcam	Ab37056	1:500	
KI67	Rbt	LSBio	LS-C141898	1:600	
NOS2	Ms	LSBio	LS-CB10049	1:400	
**Target**	**Host**	**Conjugate**	**Source**	**Catalog No.**	**Dilution**
Anti-goat	Donkey	Alexa Fluor488	Invitrogen	A11055	1:200
Anti-rabbit	Donkey	Alexa Fluor555	Life Technologies	A31572	1:200
Anti-rat	Donkey	Alexa Fluor647	Abcam	ab150155	1:200

### Confocal image analysis

Analysis of standard confocal data sets was performed by opening Leica image format files in Imaris Stitcher (v9.0, Bitplane Scientific) to fuse overlapping fields of view together. These reconstructed areas were then analyzed using Imaris software. Expression of NOS2 in IEC was determined by creating a mask based on regions of CDH1 staining (i.e. IEC) that contained DAPI^+^ cells. This defined region was then interrogated for the number of IEC present, and the intensity of NOS2 staining. Counting of DAPI^+^ Ki67^+^ IEC, or T-cells (CD3+ DAPI+) cells were performed in a similar manner in 3–5 fused fields of view from each animal counted.

### Ussing chambers

Following excision of the intestine, segments of colon were cut along the mesenteric border to allow for mounting in the Ussing chamber (Physiologic Instruments, San Diego, CA). Tissues were maintained in oxygenated Kreb’s buffer consisting (in mM) of: 115 NaCl, 1.25 CaCl_2_, 1.2 MgCl_2_, 2.0 KH_2_PO_4_ and 25 NaHCO_3_ at pH 7.35 ± 0.02 and maintained at 37°C. Additionally, glucose (10 mM) was added to the serosal buffer as a source of energy, which was balanced osmotically by mannitol (10 mM) in the mucosal buffer. Agar–salt bridges were used to monitor potential difference (PD) across the tissue, and to inject the required short‐circuit current (Isc) to maintain the PD at zero by an automated voltage clamp. Data from the voltage clamp (i.e. Isc, and PD) was continuously acquired using acquisition software (Physiologic Instruments). Baseline Isc values were obtained after equilibrium had been achieved approximately 15 min after the tissues were mounted. Isc, an indicator of active ion transport, was expressed in μA/cm^2^. After tissues reached stable short-circuit current for 15 minutes, stimulation of ion secretion was induced by addition of the muscarinic receptor agonist carbachol (20 μM). After returning to baseline the adenylate cyclase activating compound Forskolin ([FSK], 20 μM) was added and response recorded.

### *In vitro Nos2* expression

CMT-93 cell line (ATCC cat. CCL-223) from an induced carcinoma of mouse rectum was cultured in DMEM supplemented with 10% heat-inactivated fetal bovine serum (FBS supplemented with Pen Strep (ThermoFisher Scientific cat. 15070063), and maintained at 37°C in a humidified atmosphere with 5% CO_2_. Cells were seeded in a 6-well plate at a density of 1×10^6^ cell/well and incubated for 48 h. The medium was replaced with serum-free DMEM for 2 h. Then, cells were treated with IFNγ 1 ng/ml (Peprotech cat. 315–05), Carbachol (100 μM, Sigma Aldrich) or both for 3h. Cells were collected and stored at -80°C on Trizol. RNA extraction, cDNA synthesis and qPCR were performed as described above.

### Statistical analysis

Data were analyzed using one-way analysis of variance (ANOVA) in Prism (Graphpad, San Diego CA), with a P value of less than 0.05 denoted as significant.

## Results

### *C*. *rodentium* infection induces the recruitment of ChAT^+^ T-cells

**Fig 1:**
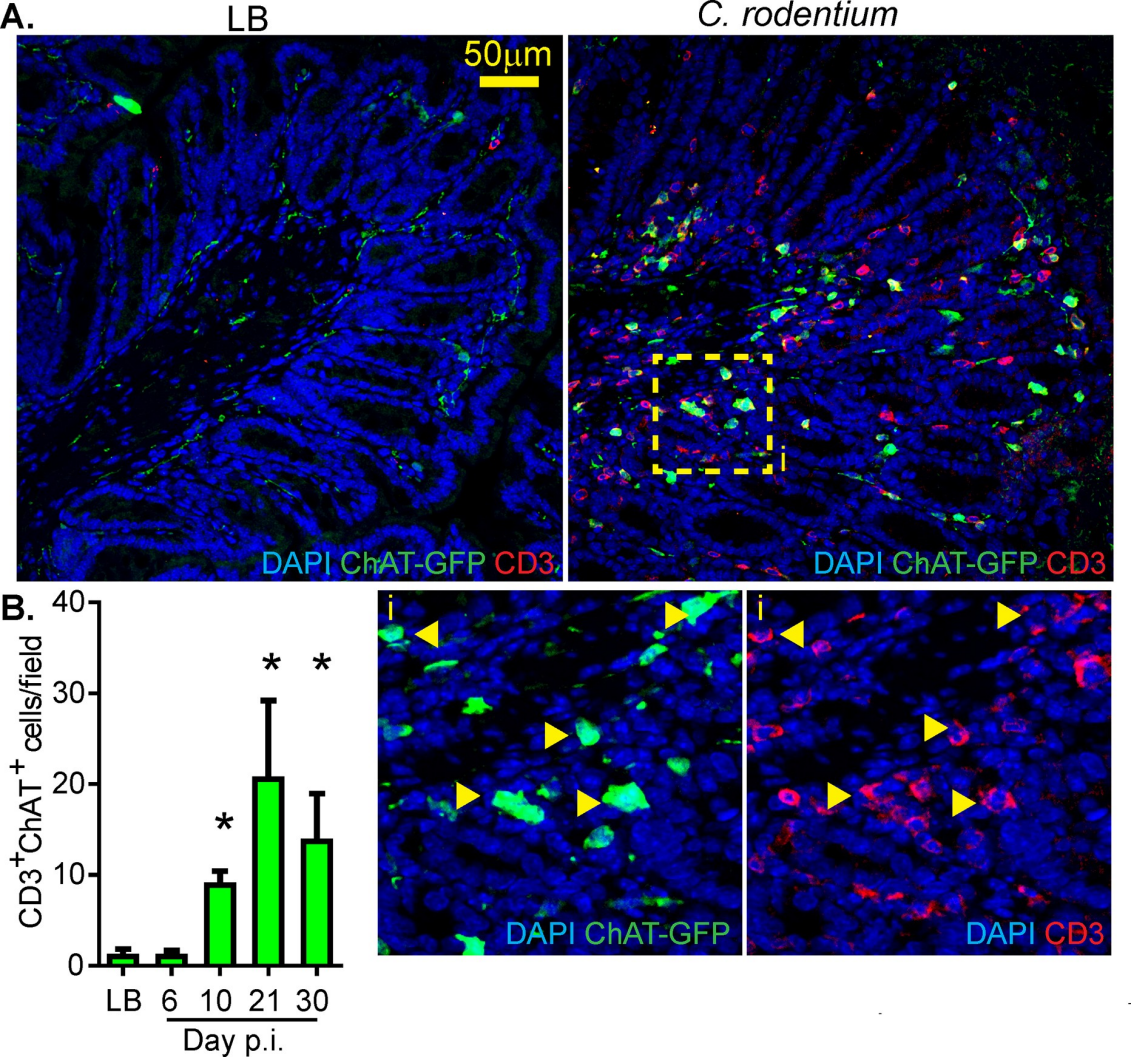
Infection with C. rodentium recruits ChAT+ T-cells to the colon. (A) Confocal microscopy was conducted on colonic tissues from LB or C. rodentium infected ChAT-GFP reporter mice using DAPI, anti-CD3, and anti-GFP. (B) These CD3+ ChAT-GFP+ cells (inset “I”) where then quantified in 5 random fields from LB control, or 6, 10, 21, or 30 days post-infection with C. rodentium. * P < 0.05 ANOVA, with n = 5–6 mice per timepoint.

**Fig 2:**
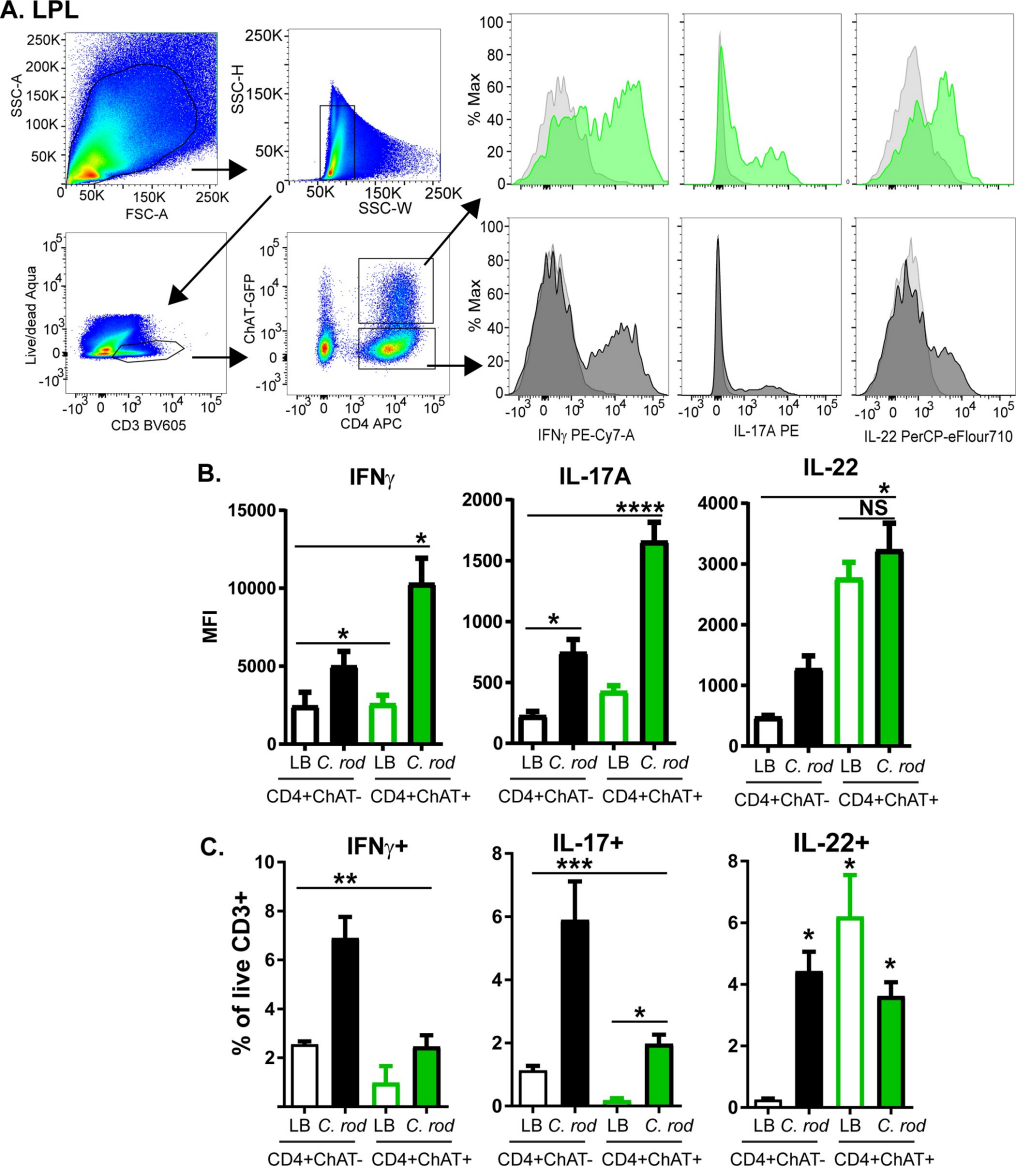
Flow cytometric characterization of ChAT-GFP+ T-cells. (A) Lamina propria lymphocytes were isolated from ChAT-GFP mice 10 days after C. rodentium infection, or (LB gavaged) controls. Representative gating strategy depicts analysis of (lymphocytes, single, cells, live CD3+). CD4+ ChAT-GFP+ or CD4+ ChAT-GFP- cells expressing IFNγ, IL-17A, or IL-22 in each of these populations (light grey: non-stimulated control, green; ChAT-GFP+ cells stimulated with PMA/ionomycin, dark grey: ChAT-GFP- cells stimulated with PMA/ionomycin). The amount of each cytokine was quantified by MFI (B), and the frequency of these cells per live CD3+ cell (C). * P < 0.05 ANOVA, with n = 4–6 mice per group.

Only sparse numbers of ChAT^+^ T-cells have been observed in the intestine of naïve mice [13], however the potential role of ChAT^+^ T-cells in the mucosal immune response during enteric bacterial infection has not been established. To assess if ChAT^+^ T-cells are recruited during infection, ChAT-GFP^+^ mice were infected with *C*. *rodentium* and the number of CD3^+^ ChAT-GFP^+^ T-cells determined by confocal microscopy on days 6, 10, 21, and 30 post-infection (p.i.). Mice infected with *C*. *rodentium* had a significant increase in the number of CD3^+^ChAT-GFP^+^ T-cells in the colon beginning 10 days p.i. which persisted until 30 days p.i. **(Fig 1A & 1B)**. In order to characterize these recruited cells, flow cytometry was conducted on isolated lamina propria lymphocytes (LPL). These colonic lamina propria ChAT-GFP^+^ T-cells (Single, Live, CD3^+^, CD4^+^) 10 days post-*C*. *rodentium* infection produced IFNγ, IL-17A, and IL-22 **(Fig 2A).** Quantification revealed that ChAT-GFP^+^ T-cells express more IFNγ, IL-17A, and IL-22 by MFI (mean fluorescence intensity) compared to ChAT-GFP^-^ T-cells **(Fig 2B).** Despite this, it is important to note that the frequency of ChAT-GFP^+^ T-cells actively producing IFNγ and IL-17A were significantly less compared to ChAT-GFP^-^ T-cells. ChAT-GFP^+^ IL-22^+^ T-cell population appears to be persistent in the naïve colon and does not increase significantly during infection. These data demonstrate that the ChAT-GFP^+^ T-cells are not unique to Th1/Th17/Th22 T-cells subsets, and can be polarized to these three different phenotypes **(Fig 2).**

In contrast to the recruitment induced by *C*. *rodentium* infection, induction of colonic inflammation by the chemical irritant DSS failed to increase the number of ChAT-GFP^+^ T-cells compared to naïve control, despite evidence of overt inflammation **(S1A Fig)**. Together, these results suggest that ChAT^+^ T-cells are a specific component of the host response to *C*. *rodentium* infection and their recruitment is driven by specific signals and rather than a simple consequence of intestinal inflammation.

### Ablation of T-cell derived acetylcholine increases colonic *C*. *rodentium*

**Fig 3:**
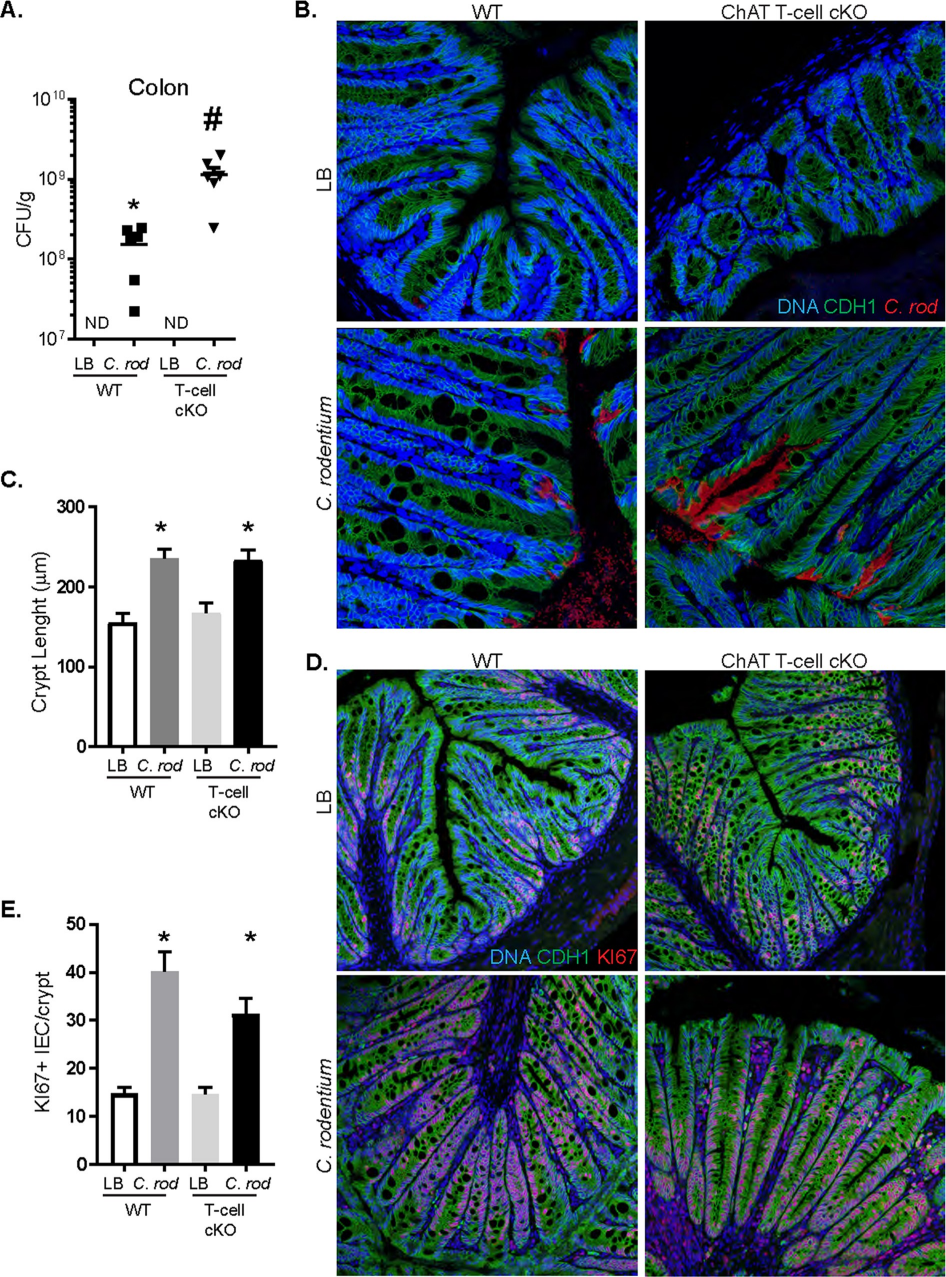
Increased C. rodentium colonization in ChAT T-cell cKO mice without increased histopathology. (A) The number of colonic tissue adherent bacteria were assessed in WT and ChAT T-cell cKO mice 10 days p.i. (B) Confocal imaging of uninfected and infected WT and ChAT T-cell cKO mice demonstrates increased adherent bacteria in the luminal surface of intestinal epithelial cells (IEC, CDH1+ DAPI+). (C) Infection of WT and ChAT T-cell cKO mice induces crypt hyperplasia as indicated by significantly increased crypt lengths. (D&E) and Ki67+ IEC compared to uninfected controls. Data are representative from 3 separate experiments with 3–5 mice per group. ND = not detected, *, # P < 0.05 ANOVA.

The functional role of T-cell-derived ACh during *C*. *rodentium* infection was determined using a T-cell conditional knockout (cKO) approach. Accordingly, infected ChAT T-cell cKO mice had increased CFU/g of *C*. *rodentium* in colonic tissue at day 10 p.i. as compared to infected WT mice **(Fig 3A)**. To determine if the increased bacterial burden of *C*. *rodentium* resulted in altered localization of the pathogen in the colon, confocal microscopy analysis using antibodies directed against *C*. *rodentium* was performed **(Fig 3B)**. Compared to WT, we observed increased *C*. *rodentium* in the colonic lumen, adjacent to IEC (CDH1^+^ DAPI^+^), and the presence of microcolonies within the colonic crypts in ChAT T-cell cKO mice. Despite the increased bacterial burden in ChAT T-cell cKO mice, no significant increase in the number of proliferating (DAPI^+^ CDH1^+^ Ki67^+^) IEC cells **(Fig 3D &3E)**, histopathological damage, or crypt hyperplasia was observed compared to infected WT mice **(Fig 3C**). Together these data indicate that T-cell derived ACh is a critical component of host defense during *C*. *rodentium* infection but does not influence epithelial barrier integrity or effect crypt hyperplasia. To assess what factors could contribute to recruitment of these CD3^+^ ChAT^+^ T-cells during *C*. *rodentium* infection, we performed qRT-PCR for chemokines that are cognate ligands for previously identified receptors expressed by this population of T-cells [4, 26]. The pattern of *Cxcl13* expression closely mirrors the recruitment of ChAT^+^ T-cells **(S2A Fig),** with significantly increased expression beginning 10 days and lasting until day 30 p.i. while significantly increased expression of *Ccl1*, *Ccl8*, *Ccl19* and *Ccl21* occurred between 21 and 30 days p.i., well after recruitment of ChAT^+^ T-cells began. Critically, infection of mice deficient in CXCR5, the cognate receptor for CXCL13, did not experience increased *C*. *rodentium* bacterial burden or pathology **(S2B Fig**). In addition, assessment of intestinal physiology using Ussing chambers revealed no significant differences in conductance, baseline or evoked short-circuit current responses to carbachol or forskolin in naïve WT or ChAT T-cell cKO mice **(S3 Fig)**.

### *C*. *rodentium*-induced inflammation is enhanced with loss of T-cell derived ACh

**Fig 4:**
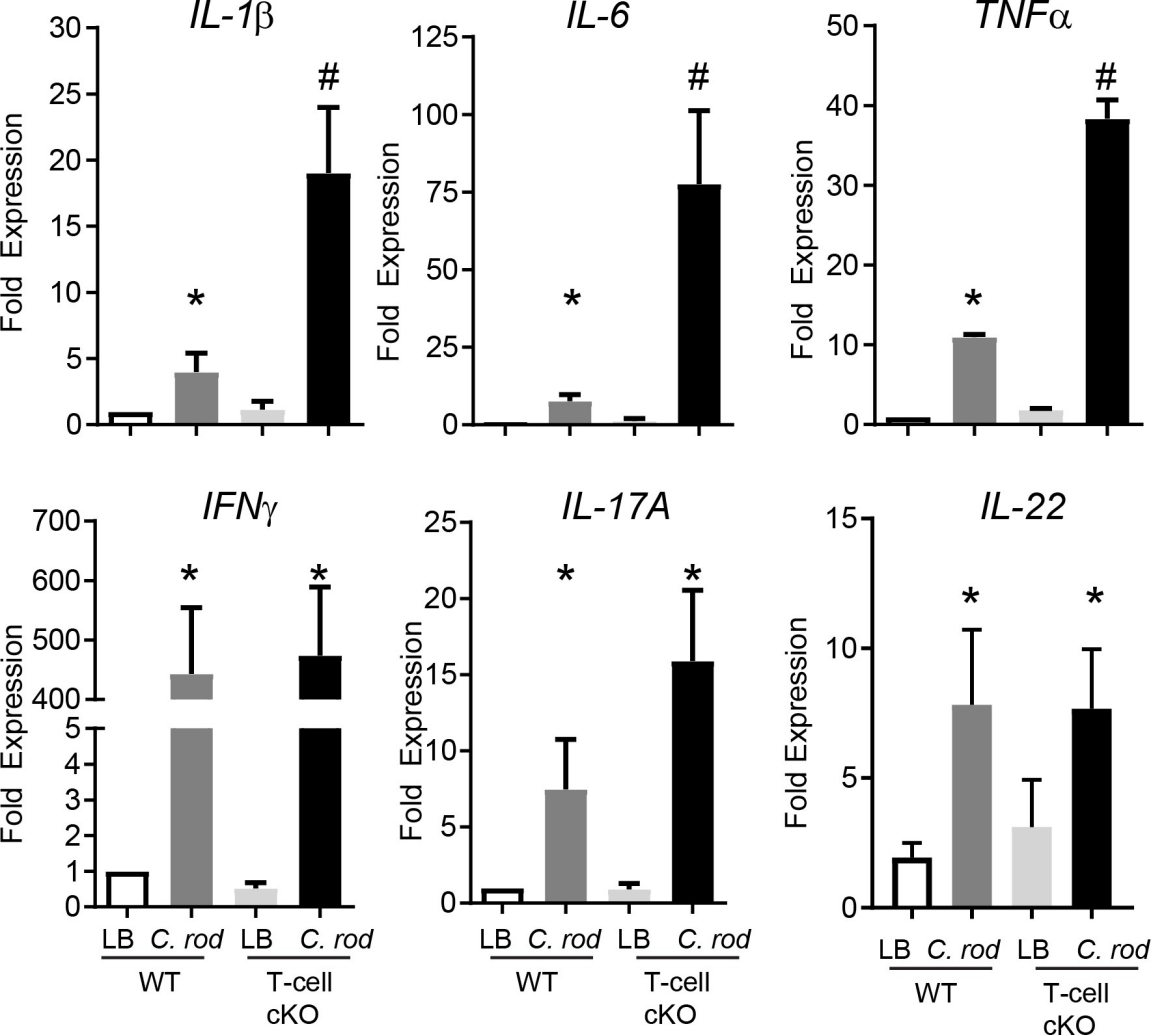
Conditional ablation of ChAT in T-cells increases C. rodentium-induced innate inflammatory genes. The immune response during infection was assessed through qRT-PCR conducted on colonic tissues from LB or C. rodentium infected WT or ChAT T-cell cKO mice. Expression of the cytokines Il1-β, Il-6, and Tnfα, Ifnγ, Il-17a and Il-22 was evaluated in naïve and infected WT and ChAT T-cell cKO mice. *, # P < 0.05 ANOVA, with n = 8–10 mice per group.

To determine the immunological consequences of conditional ablation of ChAT in T-cells during *C*. *rodentium* infection, qRT-PCR was conducted on colon from LB control and infected WT and ChAT T-cell cKO mice for proinflammatory gene expression. At day 10 p.i., expression of *Il-1β*, *Il-6*, and *Tnfα* were significantly increased in *C*. *rodentium* infected mice compared to LB control mice **(Fig 4)**. Expression of these cytokines was significantly enhanced 10 days p.i. in the ChAT T-cell cKO mice compared to WT infected animals. In contrast, expression of *Ifnγ*, *Il-17a*, *Il-22*were increased 10 days p.i. to a similar extent in WT and ChAT T-cell cKO mice (**Fig 4)**. These data indicate that ablation of ChAT in T-cells can significantly alter the host immune response to *C*. *rodentium*, but in a manner that does not alter local Th1, Th17, or Th22 responses.

### Conditional ablation of ChAT in T-cells does not affect antimicrobial peptide production

As ChAT T-cell conditional knockout mice were previously observed to have reduced expression of antimicrobial peptides [13], we questioned if this could result in an increased *C*. *rodentium* burden. Using qRT-PCR we observed no significant differences in antimicrobial peptide expression in the small intestine or colon **(S4 Fig)** in naïve WT and ChAT T-cell cKO mice. As expected, colonic expression of RegIIIγ was significantly increased after *C*. *rodentium* infection in both WT and ChAT T-cell cKO mice, however there was no difference between the two genotypes in the terminal ileum or colon **(S4A & S4B Fig)**. As the commensal microbiota actively produces bioactive metabolites, we assessed if production of short-chain fatty acids (SCFA) was different in WT compared to ChAT T-cell cKO mice. Mass spectrometry revealed significant changes in specific SCFA during *C*. *rodentium* infection. Significantly reduced lactic acid was observed in infected WT but not in ChAT T-cell cKO mice. Butyric acid was significantly enhanced in both WT and ChAT T-cell cKO infected mice compared to uninfected WT or cKO control mice. While significantly increased production of pyruvic acid was detected in the feces from uninfected ChAT T-cell cKO mice, infection reduced the concentration of this metabolite to levels observed in uninfected or C. rodentium infected control mice. **(S5 Fig)**. Together these findings indicate that the increased *C*. *rodentium* burden in ChAT T-cell cKO mice was not due to an inability to produce antimicrobial peptides or alterations in SCFA produced by the microbiota.

### T-cell derived ACh regulates host *Nos2* expression during infection

**Fig 5:**
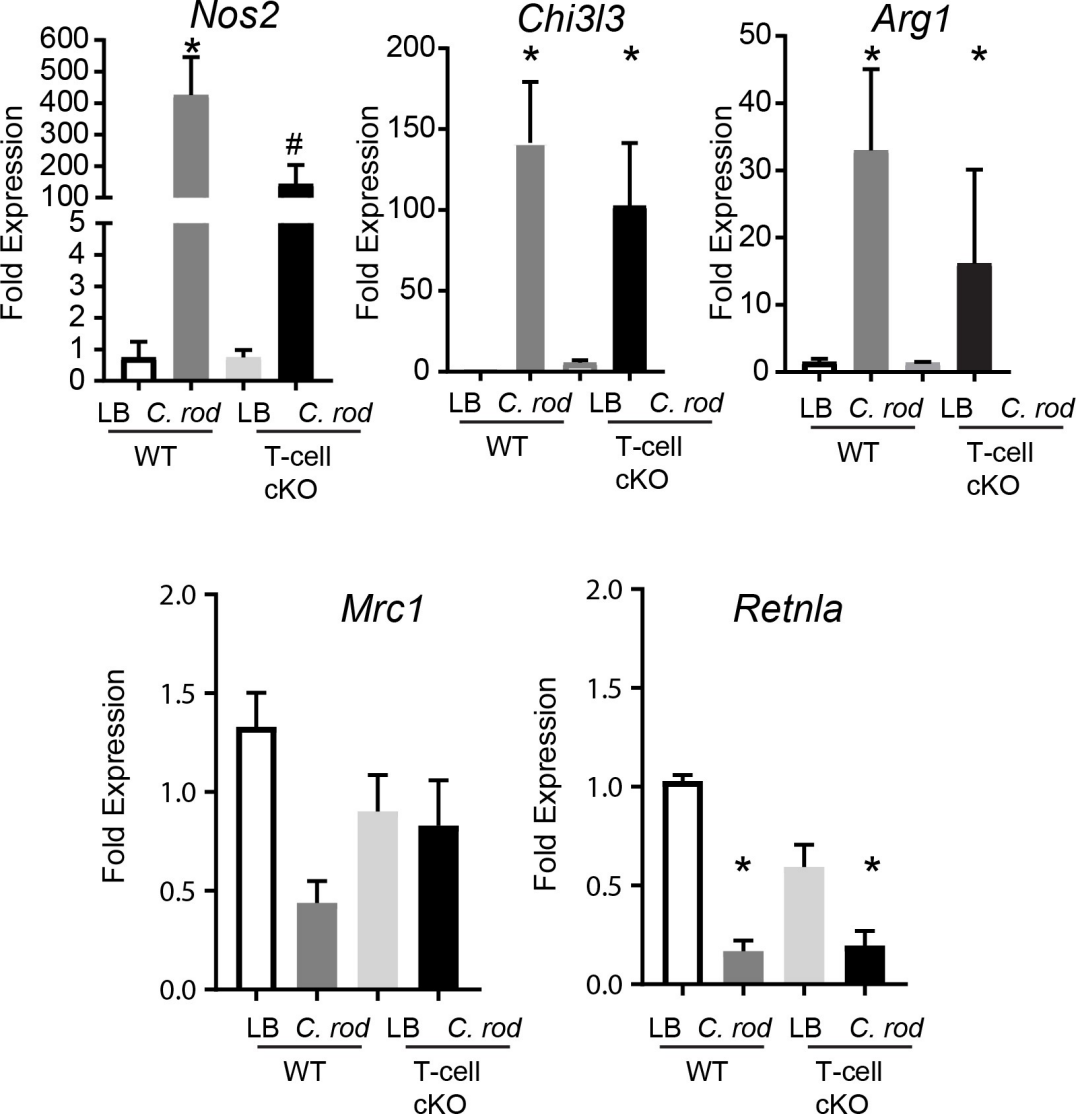
Loss of T-cell derived ACh reduces Nos2 expression during C. rodentium infection. Expression of genes characteristic of macrophage polarization were assessed by qRT-PCR on colonic tissue from LB or C. rodentium infected WT or ChAT T-cell cKO mice. *, # P < 0.05 ANOVA, with n = 8–10 mice per group.

**Fig 6:**
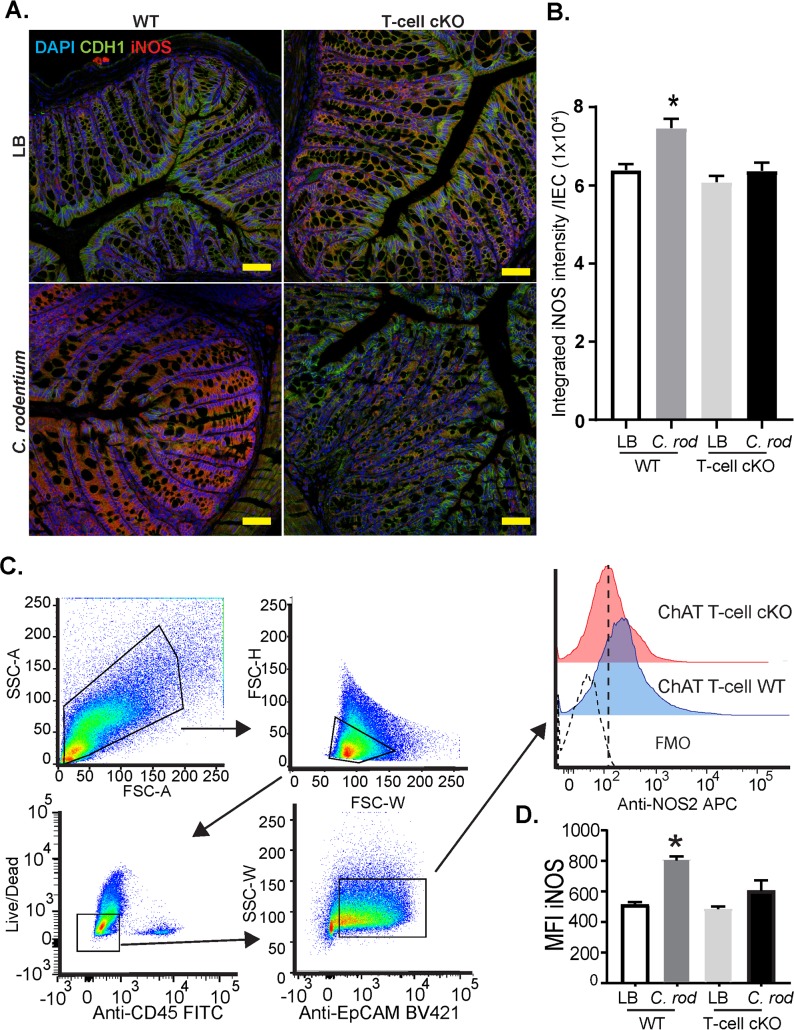
NOS2 is reduced in C. rodentium infected Intestinal epithelial cells from ChAT T-cell cKO mice. (A) Localization and quantification of NOS2 was conducted on fixed colonic tissues from LB and C. rodentium infected WT and ChAT T-cell cKO mice, 10 days p.i. Confocal microscopy was conducted using DAPI and antibodies directed against epithelial e-cadherin (CDH1), and NOS2. (B) Expression of NOS2 per IECs (DAPI+ CDH1+ cells) was then quantified using Imaris. (C) In a separate cohort of mice, NOS2 expression was evaluated 10 days p.i in isolated IEC by flow cytometry. IEC were identified as live/single/CD45- EpCAM+ cells. Expression of NOS2 was determined by MFI and is summarized in (D). *P <0.05 ANOVA n = 4–8 mice/group.

The increased expression of certain pro-inflammatory cytokines coupled with increased colonic *C*. *rodentium* burden in ChAT T-cell cKO mice lead us to ascertain if innate effector responses were intact. First, we considered if lack of T-cell derived ACh could increase differentiation of alternatively activated macrophages, disrupting the ability to mount and effect innate responses to *C*. *rodentium*. As indicated in **Fig 5**, no significant differences were noted in arginase1 (*Arg1*), mannose receptor C-type 1 (*Mrc-1*), chitinase-like 3 (*Chi3l3*), or resistin-like molecule α (*Retnla*) expression by qRT-PCR in colonic tissues between WT and ChAT T-cell cKO mice. Expression of *Nos2* (“iNOS”) however was significantly abrogated 10 days p.i. in ChAT T-cell cKO mice compared to infected WT. These data indicate that lack of T-cell derived ACh does not increase alternatively activated/M2 macrophage polarization, but significantly impacts the expression of *Nos2*. As numerous cell types can express NOS2, we assessed the localization and quantity of NOS2 protein by confocal microscopy on colonic tissue from infected WT and ChAT T-cell cKO mice and uninfected controls. As indicated in **Fig 6**, IEC (CDH1^+^ DAPI^+^) were the predominant cell type that were immunoreactive of NOS2 during *C*. *rodentium* infection. In keeping with the qRT-PCR data, *C*. *rodentium* induced NOS2 expression was significantly reduced in ChAT T-cell cKO compared to WT mice (**Fig 6A)**. Quantification of NOS2 expression in IEC further demonstrate reduced NOS2 expression in *C*. *rodentium* infected ChAT T-cell cKO mice **(Fig 6B)**. This reduced ability to increase NOS2 expression in IEC during 10 days p.i. was further validated by flow cytometry conducted on IEC (Single, live, CD45^-^, EpCAM^+^) from naïve and infected WT and ChAT T-cell cKO mice **(Fig 6C & 6D).** These data demonstrate that T-cell deficiency in ChAT significantly impairs *C*. *rodentium* induced increases of NOS2 expression in IEC.

### Acetylcholine enhances expression of NOS2 in IEC

**Fig 7:**
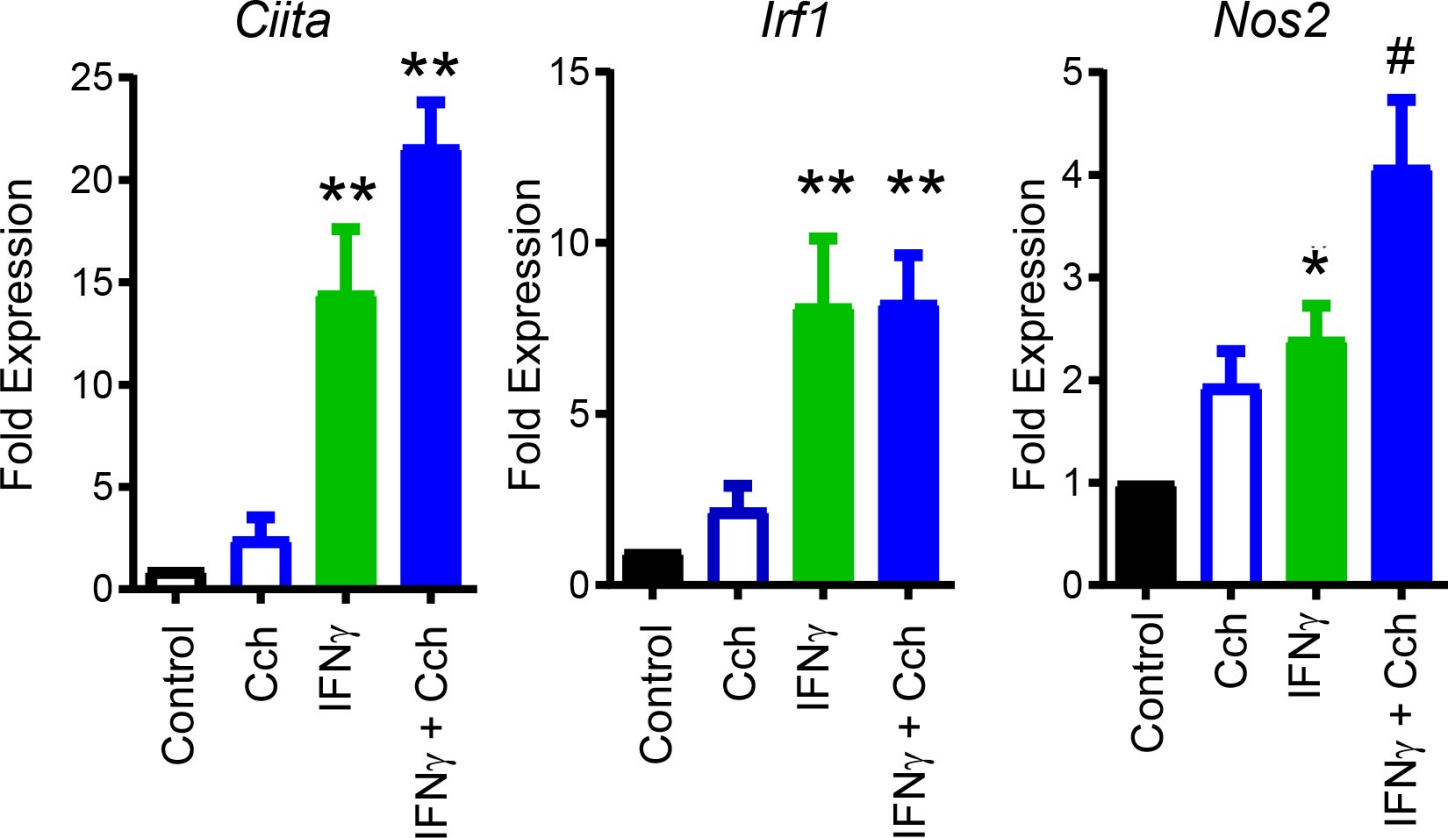
Nos2 expression induced by IFNγ in IEC is enhanced by cholinergic agonists. CMT-93 cells were stimulated with vehicle (control), Carbachol (‘Cch’ 100 μM), IFNγ (1 ng/mL), or IFNγ (1ng/mL) + Cch (100 μM) for 3h. Expression of IFNγ-induced genes Ciita, Irf1 and Nos2 was quantified by qRT-PCR. *, # P<0.05, **P < 0.01, ANOVA, n = 4 independent experiments.

As ACh has been previously demonstrated to induce NOS2 expression in lung epithelial cells [27], we sought to determine if ACh could induce similar effects in IEC. The mouse colonic epithelial cell line CMT-93 was treated with IFNγ ± carbachol (ACh mimetic), with *Nos2*, *irf1*, *and CIIta* expression assessed by qRT-PCR. As expected, stimulation with IFNγ (1 ng/mL, 3 h, time and dose determined empirically) induced expression of *Ciita*, *Irf1*, and *Nos2*. Co-treatment with carbachol further significantly increased expression of *Nos2* compared to IFNγ alone, but did not enhance *Ciita* or *Irf1* expression **(Fig 7).** Treatment with carbachol alone failed to significantly increase expression of any of the target genes. These results suggest that cholinergic signaling in IEC can synergistically enhance select IFNγ induced genes including *Nos2*.

## Discussion

The finding that the nervous system is an active participant during inflammation has been an unexpected and intriguing finding. At the interface between these two systems is a unique type of T-cells that can produce and release ACh in response to sympathetic neurotransmitters [2, 28]. Although the predominant focus on these ChAT^+^ T-cells has been on their ability to reduce the severity of disease in a number of clinically relevant immunopathologies [5–9], ChAT^+^ T-cells can also help to establish host-commensal interactions. While a prior study demonstrated increased diversity of the small intestinal microbiota in ChAT T-cell cKO mice, due to reduced antimicrobial peptide expression [13], the role of these cells during enteric bacterial infection was not known.

Using a combination of ChAT-GFP reporter and ChAT T-cell cKO mice, our studies are the first to demonstrate recruitment of ChAT^+^ T-cells and a functional role for these cells during *C*. *rodentium* infection. These recruited ChAT-GFP^+^ T-cells do not appear to be restricted to a unique Th subset, with ChAT-GFP^+^ T-cells found to produce IFNγ, IL-17A, or IL-22 in agreement with prior studies [3].

As we and others have previously demonstrated that ChAT^+^ T-cells express the chemokine receptors CXCR5 [4] and CCR8 [29]; we sought to characterize the production of cognate ligands to these receptors during *C*. *rodentium* infection, *Cxcl13* and *Ccl8* respectively. Our analysis indicates that *Cxcl13* but not *Ccl8* expression is induced beginning 10 days p.i. until day 30 p.i., a period during infection that closely mirrors when the number of ChAT^+^ T-cells increased in the colon. This temporal pattern of chemokine expression is corroborated by other studies demonstrating increased *Ccl8* during *C*. *rodentium* infection [30]. CXCL13 is well established as critical to organization of secondary lymphatic organs [31], tertiary lymphoid tissues and recruitment of IL-22 producing ILC3 [32] and can be induced by vagal nerve stimulation [33]. Despite this, our studies using CXCR5 KO mice indicate that this signaling axis is either not critical or functionally redundant with respect to the host response to *C*. *rodentium* 10 days post-infection.

The importance of ACh derived from T-cells to host mucosal immune response during enteric bacterial infection was determined using a ChAT T-cell conditional knockout. Highlighting the host protective role of ACh, we observed an increased bacterial burden following enteric *C*. *rodentium* infection in ChAT T-cell cKO compared to WT mice. This increased bacterial burden in ChAT T-cell cKO mice was associated with significantly increased expression of the pro-inflammatory cytokines *Il-1β*, *Il-6*, *Tnfα*, with equivalent expression of *Ifnγ*, *Il-17a*, or *Il-22* compared to infected WT mice. Loss of T-cell derived Ach however did not impinge on IL-22 production, typically produced by ILC or T-cells in response to *C*. *rodentium* infection [34]. Together these findings, supported by the literature, suggest that ChAT^+^ T-cells are important in eliciting host-protective responses.

Mucosal immunity is comprised of a multitude of overlapping mechanisms that serve to protect the host from pathogens including the production and secretion of antimicrobial peptides. T-cell derived ACh has been implicated in regulation of host-microbial interactions at the mucosal surface by controlling antimicrobial peptide production. Conditional ablation of ChAT in CD4^+^ cells using CD4.Cre ChAT^f/f^ mouse line resulted in reduced *lysozyme*, *defensin A*, and *ang4* expression in the small intestine, consequently increasing the diversity of commensal microbiota in the jejunum but not the cecum, or colon [13]. In contrast to these findings we noted no significant reductions in antimicrobial peptide expression in ChAT T-cell cKO compared to WT mice. As expected [35], expression of RegIIIy was significantly enhanced in WT and ChAT T-cell cKO mice during *C*. *rodentium* infection irrespective of genotype. These data suggest that the increased bacterial burden in ChAT T-cell cKO mice was not due to a deficit in antimicrobial peptide expression.

Host production of free radicals including NO are critical factors in protection against several bacterial pathogens [36–38]. NO also functions as a short-lived cell signaling molecule and is produced by three distinct isoforms of nitric oxide synthase that are each uniquely regulated in a tissue- or context-dependent manner [37]. In contrast to the constitutively expressed NOS found in endothelium or neurons, bacterial products or inflammation can induce NOS2 expression in a variety of cell types [37, 39]. Infection with *C*. *rodentium* increases NOS2 expression, functioning to limit bacterial burden and disease [14, 40]. In agreement with this literature, our data demonstrate that IEC in the colon are the predominant cell type expressing NOS2 during *C*. *rodentium* infection in WT mice. Conditional ablation of ChAT in T-cells, however, resulted in significantly reduced *Nos2* expression compared to WT mice. Confocal microscopy on colonic tissue sections and flow cytometry experiments confirmed NOS2 expression was significantly increased in colonic IEC of WT mice, but not in ChAT T-cell cKO mice during infection. Together these data demonstrate that lack of T-cell derived ACh significantly reduced the induction of NOS2 in IEC during *C*. *rodentium* infection. Although NOS2 expression is characteristically elicited by IFNγ-induced activation of STAT1-dependent gene transcription [41], expression of this cytokine was not affected by ChAT T-cell deficiency. Additionally, we observed that acetylcholine mimetics significantly enhance IFNγ-induced *Nos2* expression in IEC *in vitro*, in agreement with previously reported experiments in lung epithelial cells [27].

There are striking similarities in the aberrant host response to *C*. *rodentium* infection in ChAT T-cell cKO and the previously described *Nos2*^*-/-*^ mice [14]. For example, both mouse lines exhibit increased bacterial burden at day 10 p.i. without resulting in increased mortality or enhanced colonic histopathology [14]. Although *Nos2* deficiency in mice, or inhibition of NO production increases Th17 differentiation [42], no significant increase in *Il-17a* expression was observed in the colonic tissue from *C*. *rodentium* infected ChAT T-cell cKO mice compared to WT mice. This is likely due to the short half-life of NO in biological fluids [43], and the expression of NOS2 in colonic IEC far from differentiating T-cells in draining lymph nodes.

Our data further substantiate the unique role of ACh producing ChAT^+^ T-cells in modulating immune function. These unique T-cells appear to function as a critical component of the mucosal immune system, limiting the number and detrimental effects of enteric bacterial pathogens. How these specialized T-cells that are recruited to the colon, become activated, and release ACh during *C*. *rodentium* infection warrants future study. Given the requirement for NE signaling through the β2AR receptor on ChAT^+^ T-cells in septic shock [2], activation by the sympathetic innervation is a strong possibility. Supporting this contention, *Salmonella typhimurium* induces activation of the sympathetic innervation within the small intestine, and the release of NE adjacent to muscularis macrophages [44]. While it is tempting to speculate that infection induced activation of a neuronal circuit is host protective, it is important to note that host NE induces bacterial expression of virulence genes by enteric pathogens such as *C*. *rodentium* [45] and enterohemorrhagic *Escherichia coli* [46]. Future studies will only further illuminate the integrated nature of the nervous system and immune system with ChAT^+^ T-cells as a critical node mediated host protection during enteric bacterial infection.

## Supporting information

S1 Fig Recruitment of ChAT+ T-cells does not occur during chemically induced colitis. **(A)** Recruitment of ChAT-GFP^+^ T-cells during intestinal inflammation was assessed following administration of DSS (day 8, 3% w/v) or infection *C*. *rodentium* (day 10 post-infection). DSS administration resulted in clinical pathology as evidence by **(B)** weight loss, **(C)** colonic shortening and **(D)** increased macroscopic disease score. * P<0.05 Student’s two-tailed T-test, n = 5–8 mice per group.
(TIF)

S2 Fig *C. rodentium* host responses are CXCR5 independent. **(A)** Full thickness colonic tissues were subjected to qRT-PCR analysis for expression of *Cxcl13*, *Ccl1*, *Ccl8*, *Ccl19*, *and Ccl21*. # P < 0.001, * P < 0.05 ANOVA, with n = 5–6 mice per timepoint. **(B)** The importance of the CXCL13-CXCR5 recruitment axis was assessed in WT and CXCR5 KO mice by CFU at day 10 p.i., and colonic hyperplasia. * P<0.05, ANOVA, n = 6–9 mice per group.
(TIF)

S3 Fig Colonic physiology is not altered in ChAT T-cell cKO mice. Ussing chambers were used to assess conductance, baseline short-circuit current, and responses to carbachol and forskolin (n = 8 mice per group).
(TIF)

S4 Fig Analysis of antimicrobial peptide production. Expression of antimicrobial peptides were assessed by qRT-PCR in the terminal ileum **(A)**, or colon **(B)** of naïve or *C*. *rodentium* infected WT and ChAT T-cell cKO mice, n = 8–10 mice per group.
(TIF)

S5 Fig Analysis of fecal short-chain fatty acids ChAT T-cell cKO mice. Fecal pellets were obtained from naïve and infected WT and ChAT T-cell cKO mice and analyzed for SCFA by mass spectrometry. Results are expressed as ng/mg of feces, n **=** 5–8 mice per group, *P<0.05, ANOVA.
(TIF)
