# Supplementary figures and images for: T-cell derived acetylcholine aids host defenses during enteric bacterial infection with Citrobacter rodentium
Source: PLoS Pathog. 2019 Apr 11;15(4):e1007719. doi: 10.1371/journal.ppat.1007719 (PMC6478367; doi:10.1371/journal.ppat.1007719)

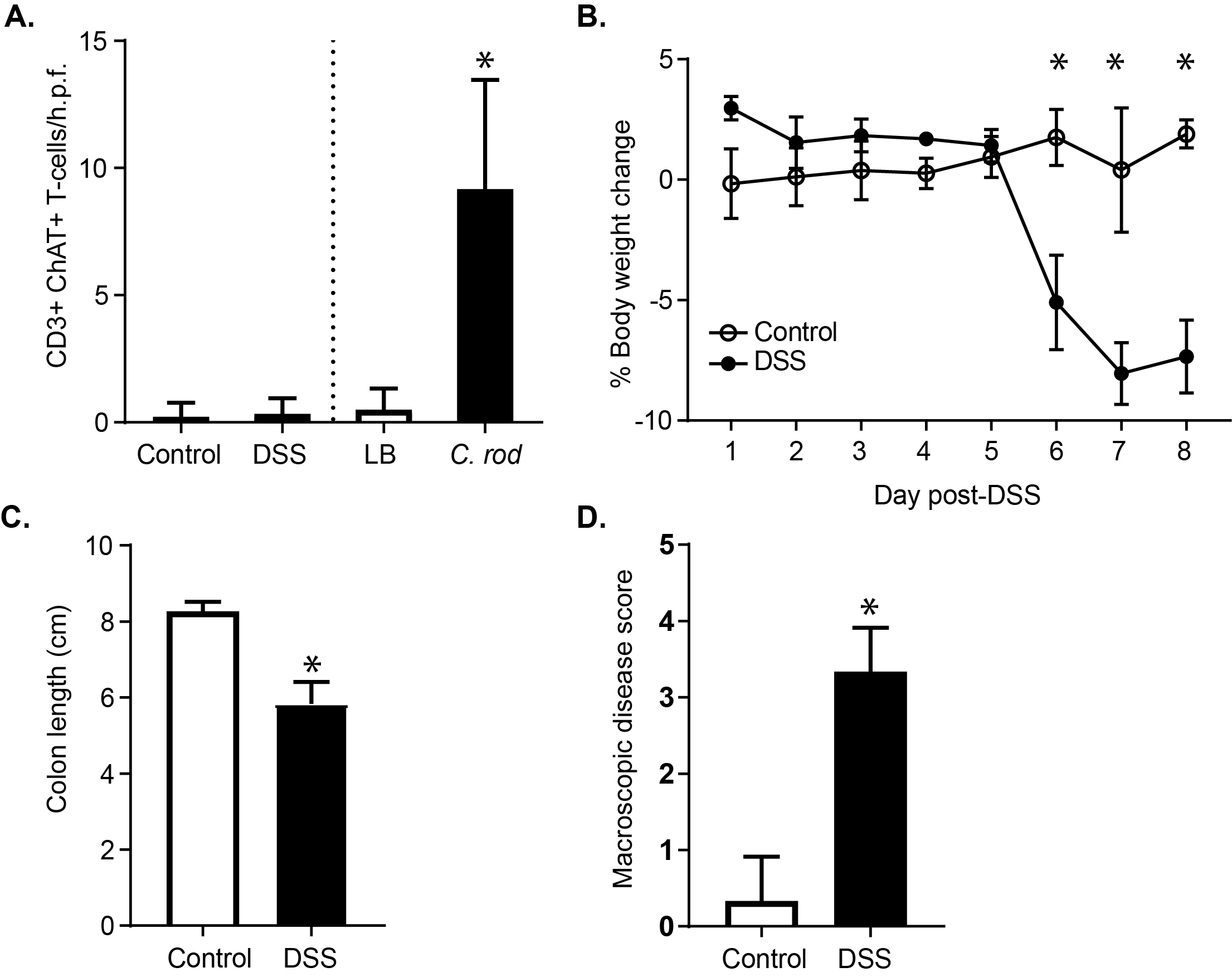

Supplement: S1 Fig — (A) Recruitment of ChAT-GFP+ T-cells during intestinal inflammation was assessed following administration of DSS (day 8, 3% w/v) or infection C. rodentium (day 10 post-infection). DSS administration resulted in clinical pathology as evidence by (B) weight loss, (C) colonic shortening and (D) increased macroscopic disease score. * P<0.05 Student’s two-tailed T-test, n = 5–8 mice per group. (TIF) [file ppat.1007719.s001.tif]

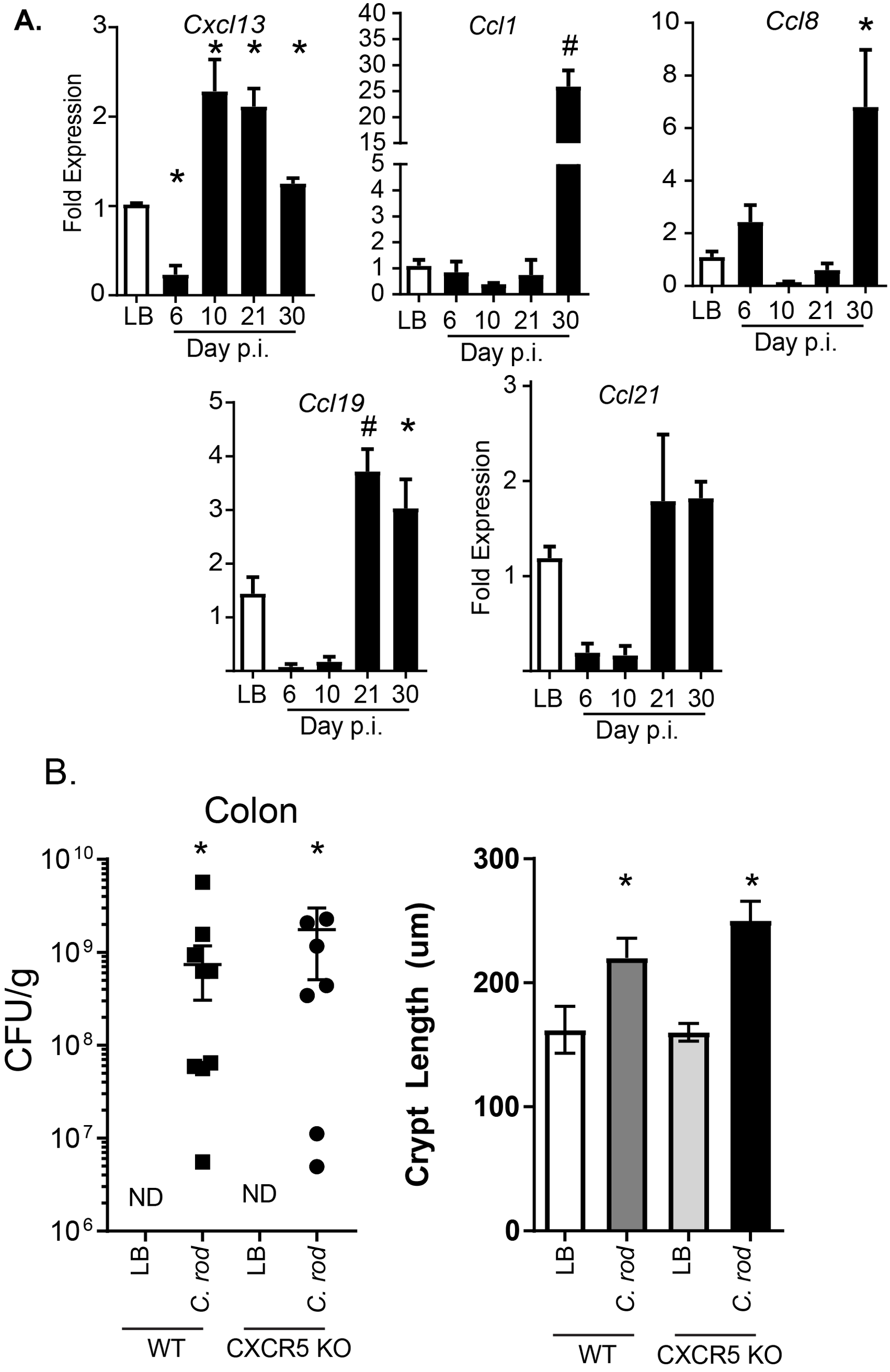

Supplement: S2 Fig — (A) Full thickness colonic tissues were subjected to qRT-PCR analysis for expression of Cxcl13, Ccl1, Ccl8, Ccl19, and Ccl21. # P < 0.001, * P < 0.05 ANOVA, with n = 5–6 mice per timepoint. (B) The importance of the CXCL13-CXCR5 recruitment axis was assessed in WT and CXCR5 KO mice by CFU at day 10 p.i., and colonic hyperplasia. * P<0.05, ANOVA, n = 6–9 mice per group. (TIF) [file ppat.1007719.s002.tif]

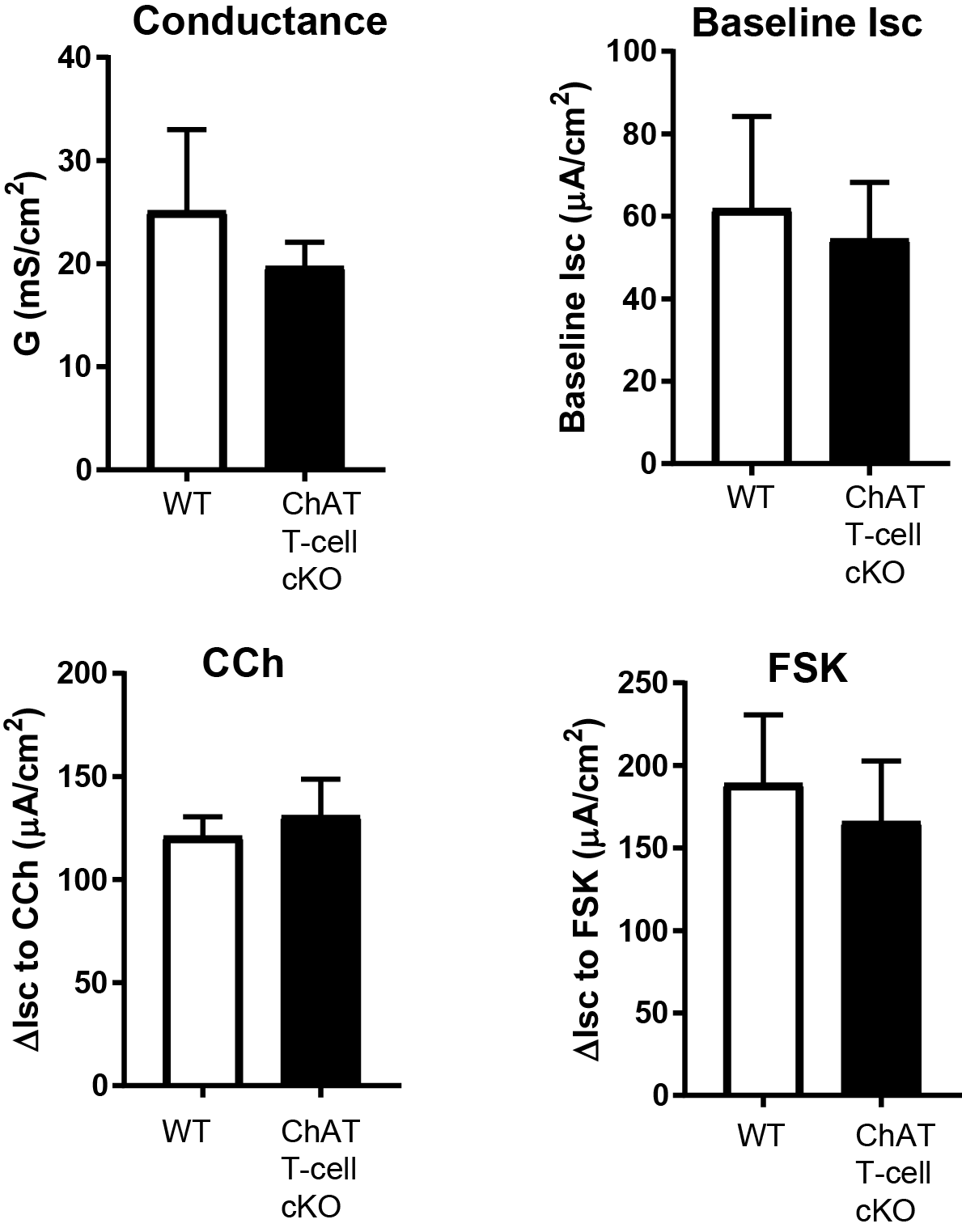

Supplement: S3 Fig — Ussing chambers were used to assess conductance, baseline short-circuit current, and responses to carbachol and forskolin (n = 8 mice per group). (TIF) [file ppat.1007719.s003.tif]

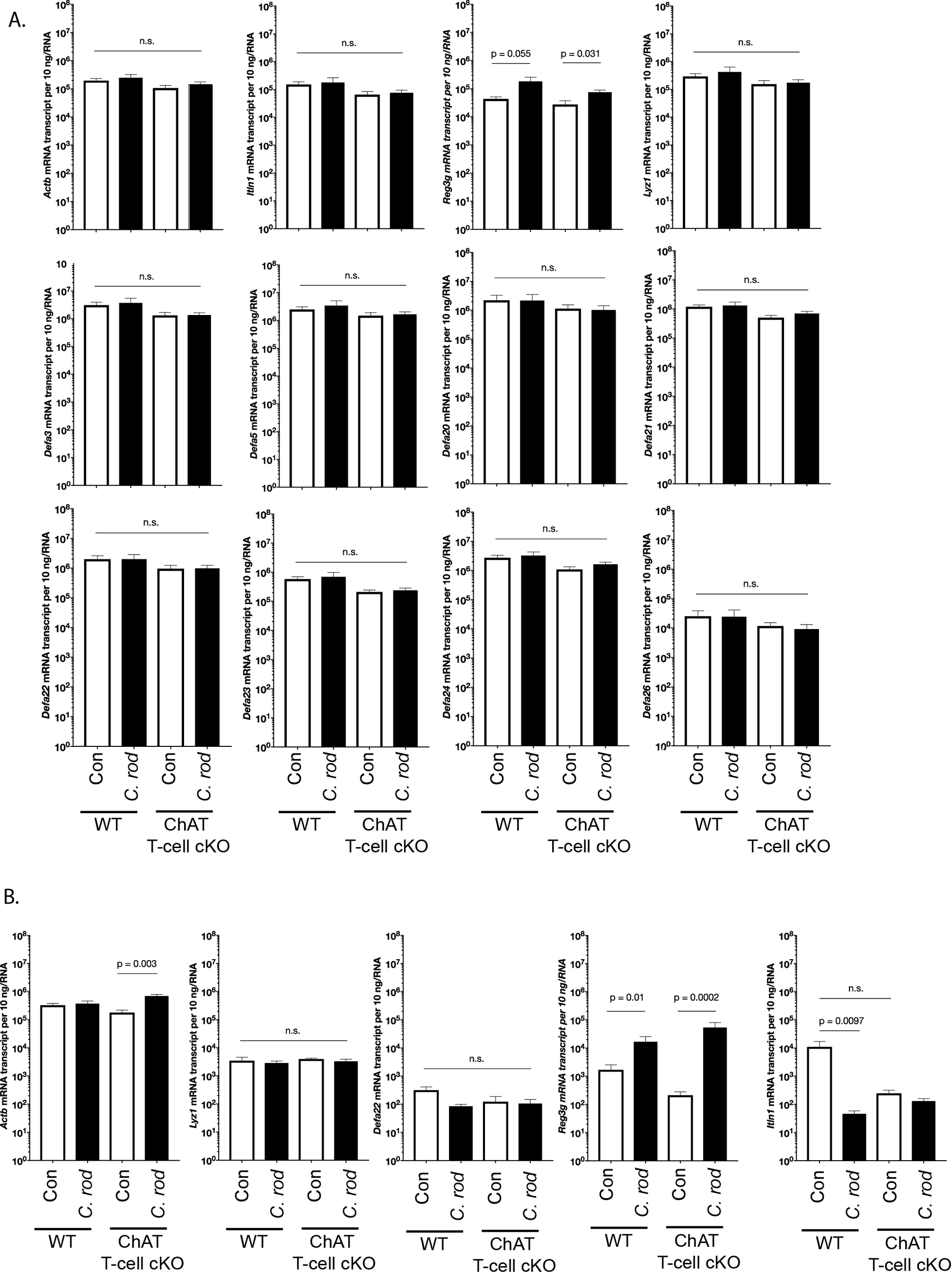

Supplement: S4 Fig — Expression of antimicrobial peptides were assessed by qRT-PCR in the terminal ileum (A), or colon (B) of naïve or C. rodentium infected WT and ChAT T-cell cKO mice, n = 8–10 mice per group. (TIF) [file ppat.1007719.s004.tif]

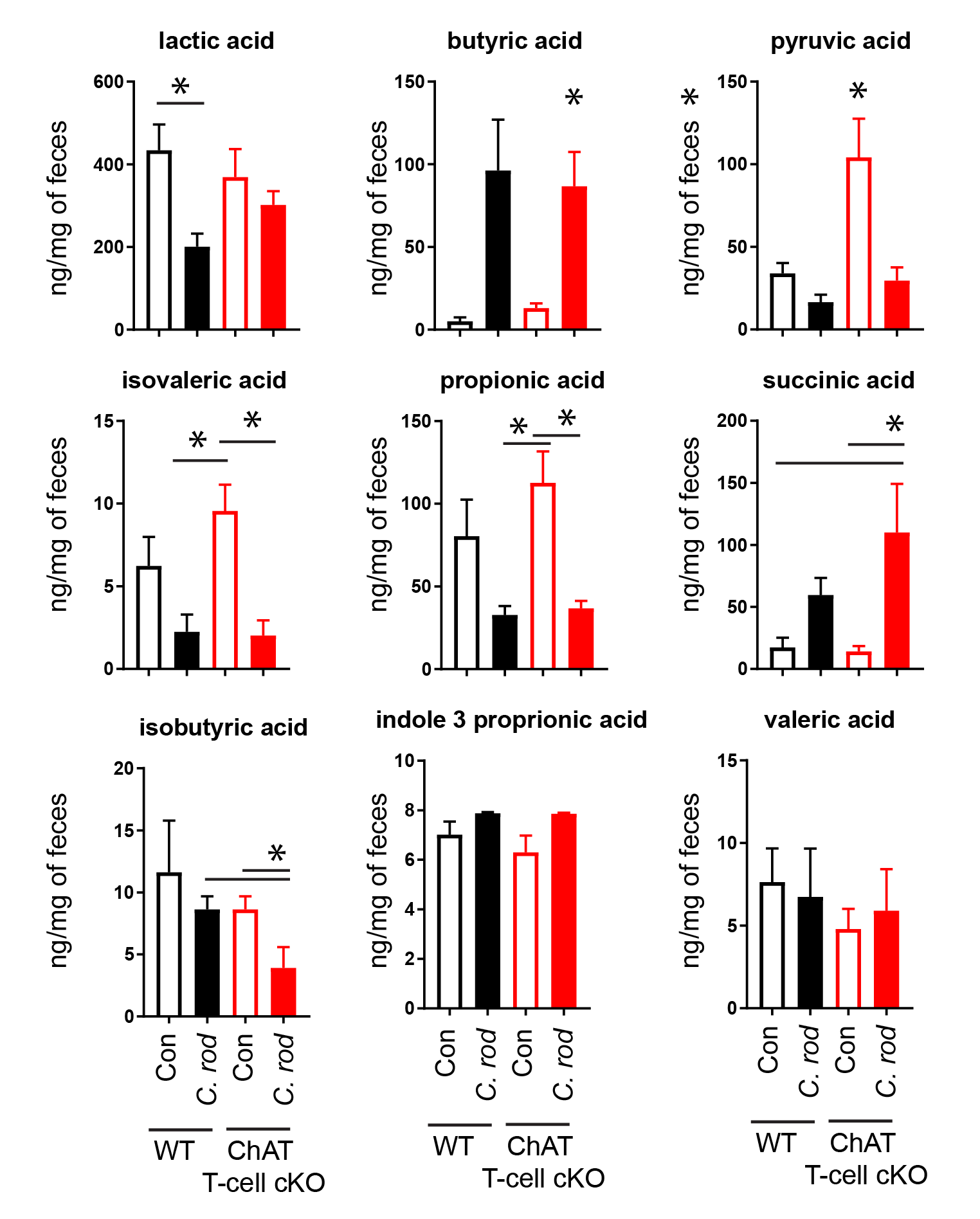

Supplement: S5 Fig — Fecal pellets were obtained from naïve and infected WT and ChAT T-cell cKO mice and analyzed for SCFA by mass spectrometry. Results are expressed as ng/mg of feces, n = 5–8 mice per group, *P<0.05, ANOVA. (TIF) [file ppat.1007719.s005.tif]
